# Supplementary material for: Genes for highly abundant proteins in Escherichia coli avoid 5’ codons that promote ribosomal initiation
Source: PLoS Comput Biol. 2023 Oct 25;19(10):e1011581. doi: 10.1371/journal.pcbi.1011581 (PMC10599525; doi:10.1371/journal.pcbi.1011581)

Fig S1 V_{edIO} does not predict patterns of codon usage at the 5' ends of genes with highly abundant proteins in four different bacterial genomes: A: *Microcystis aeruginosa*, **B**: *Mycobacterium tuberculosis*, **C**: *Bacillus subtilis*, **D**: *Pseudomonas aeruginosa*. In each panel, . The *x* axis is the log odds ratio for the codon being associated with high protein expression levels when used at the 5'-end in experimental transgenes (edIO). The *y* axis is the log odds ratio for the codon being enriched at the 5'-ends of genes with high protein abundance compared to low protein abundance in the relevant bacteria. Each data point is labelled as the codon it represents. Principle Components Analysis (PCA) was used to fit orthogonal regression lines. The Pearson's correlation coefficient and p-value are provided within each panel. It was noted that these correlations were obtained despite the experimental data coming from *E. coli* and the proteomics data from other species.

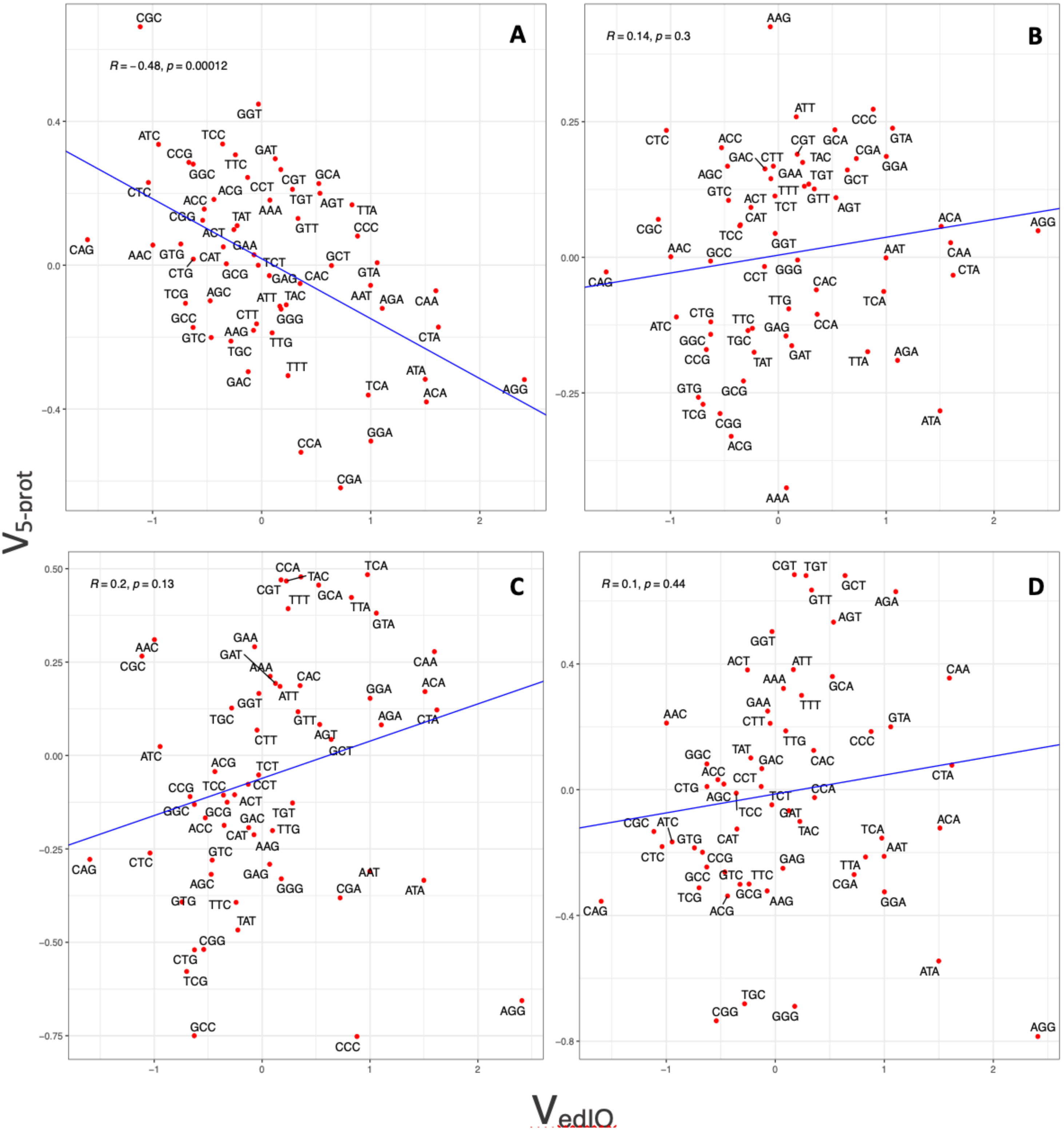

Supplement: S1 Fig — (PDF) [file pcbi.1011581.s005.pdf]
