# Supplementary material for: Genes for highly abundant proteins in Escherichia coli avoid 5’ codons that promote ribosomal initiation
Source: PLoS Comput Biol. 2023 Oct 25;19(10):e1011581. doi: 10.1371/journal.pcbi.1011581 (PMC10599525; doi:10.1371/journal.pcbi.1011581)

Fig S2 The relationship between translational optimality scores and initiation optimal scores. A. The *x* axis is the log odds ratio for the codon being enriched at the core of native genes with high protein abundance compared to low protein abundance. These values correlate well with the original w scores and Sharp and Li (rho = 0.9, P<2 x 10^{-16}). The *y* axis is the log odds ratio for the codon being enriched at the 5'-ends of transgenes with high protein abundance. Each data point is labelled as the codon it represents. **B.** As for figure A, but comparing all pairwise combinations of synonymous codons (i.e. within the same codon block: N=87). The pairwise differences are oriented such that, on the x axis, the codon with the lower value of the log odds ratio has its value subtracted from that of the higher value. The orientation is preserved for the y axis. This way no values on the x axis are negative. Each point is labelled by the oriented codon pair (first codon in the pair has the higher x-axis value, as seen in the A figure). For both figures, Principle Components Analysis (PCA) was used to fit an orthogonal regression line. The Pearson's correlation coefficient and p-value are provided within the figures.

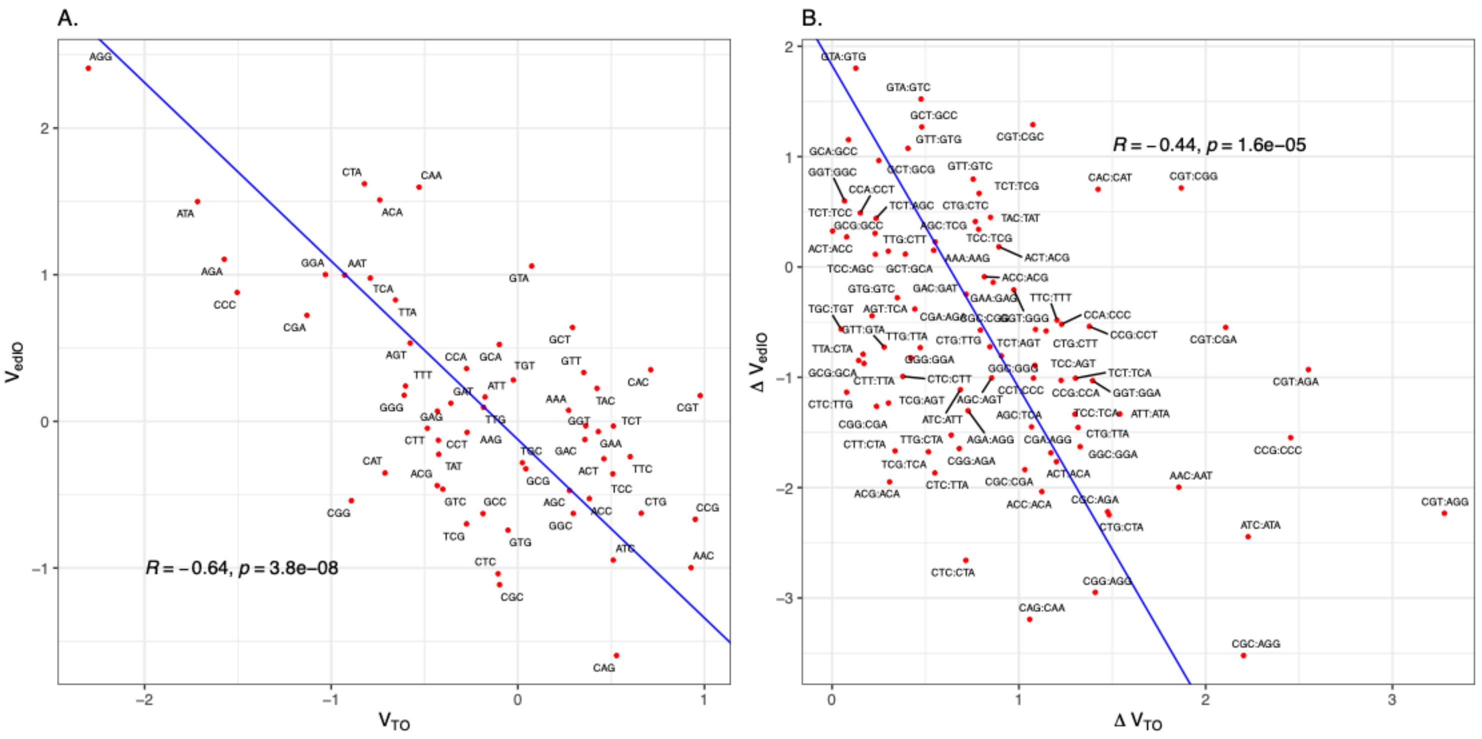

Supplement: S2 Fig — (PDF) [file pcbi.1011581.s006.pdf]
