# Supplementary material for: Genes for highly abundant proteins in Escherichia coli avoid 5’ codons that promote ribosomal initiation
Source: PLoS Comput Biol. 2023 Oct 25;19(10):e1011581. doi: 10.1371/journal.pcbi.1011581 (PMC10599525; doi:10.1371/journal.pcbi.1011581)

Fig S4. The relationship between GC3 and the difference in predicted stability of the 5' ends and core sections of all genes in each of 650 genomes. A positive difference indicates a lower stability of 5' end compared to the core.

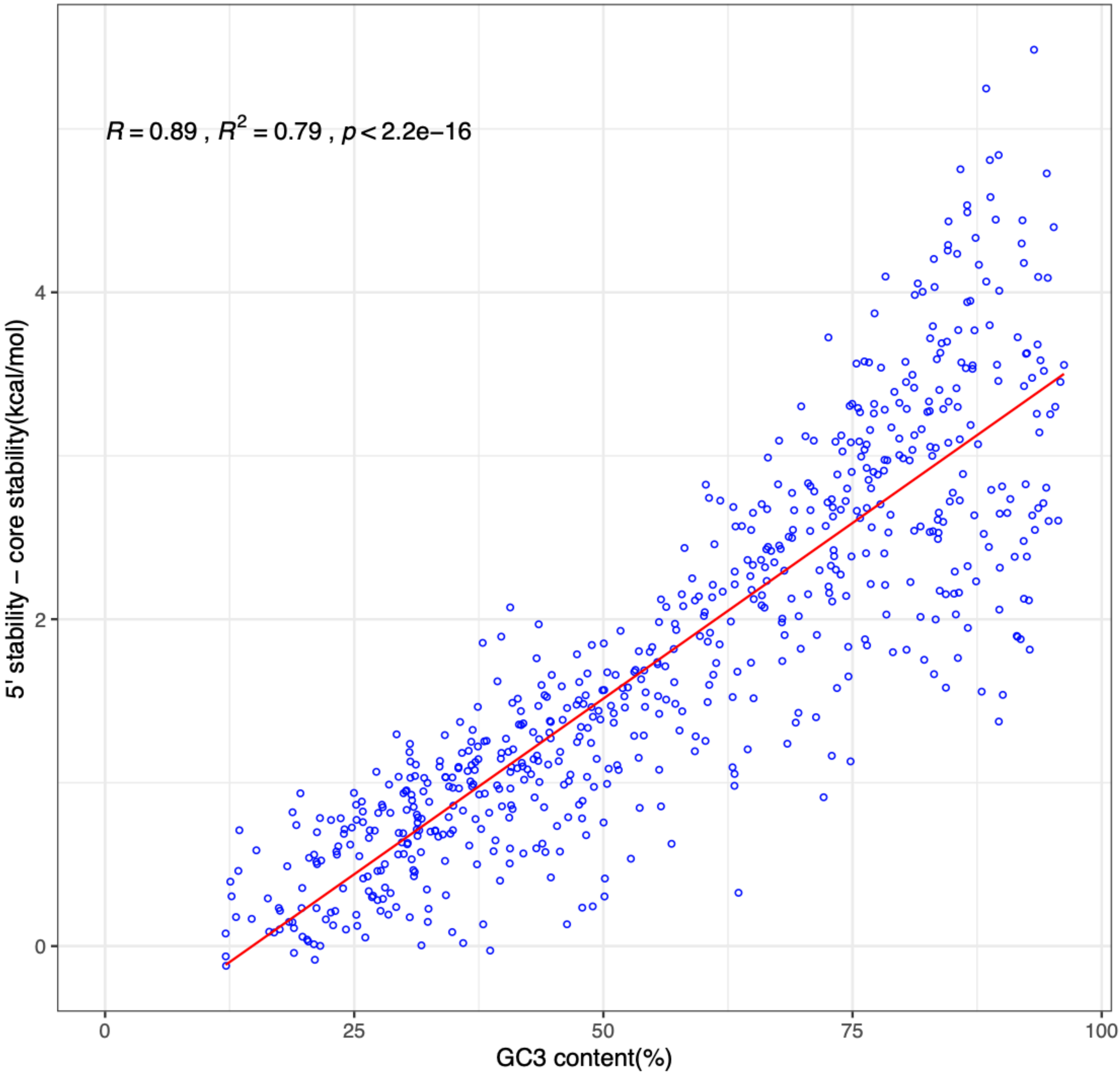

Supplement: S4 Fig — (PDF) [file pcbi.1011581.s008.pdf]
