# Supplementary material for: Genes for highly abundant proteins in Escherichia coli avoid 5’ codons that promote ribosomal initiation
Source: PLoS Comput Biol. 2023 Oct 25;19(10):e1011581. doi: 10.1371/journal.pcbi.1011581 (PMC10599525; doi:10.1371/journal.pcbi.1011581)
Supplement: S5 Fig — The E. coli genes are rank ordered by their protein abundance (expression level). They are then split into overlapping bins of various sizes (1/4 the total, 1/5th the total etc). For each bin of genes a 5’ enrichment score (compared to core) is calculated for each codon. This vector is then compared with VedIO the y axis being the correlation between the two. The horizontal dashed line is the comparable correlation considering all genes. The bins are ordered left (lowest expression) to right (highest expression bin). The x axis value is the rank order percentile position of the lowest expressed gene in the given bin. Note that correlation is lowest for both the most highly expressed and most lowly expressed genes. Intermediate bins give correlations in excess of r = 0.8. (PDF) [file pcbi.1011581.s009.pdf]

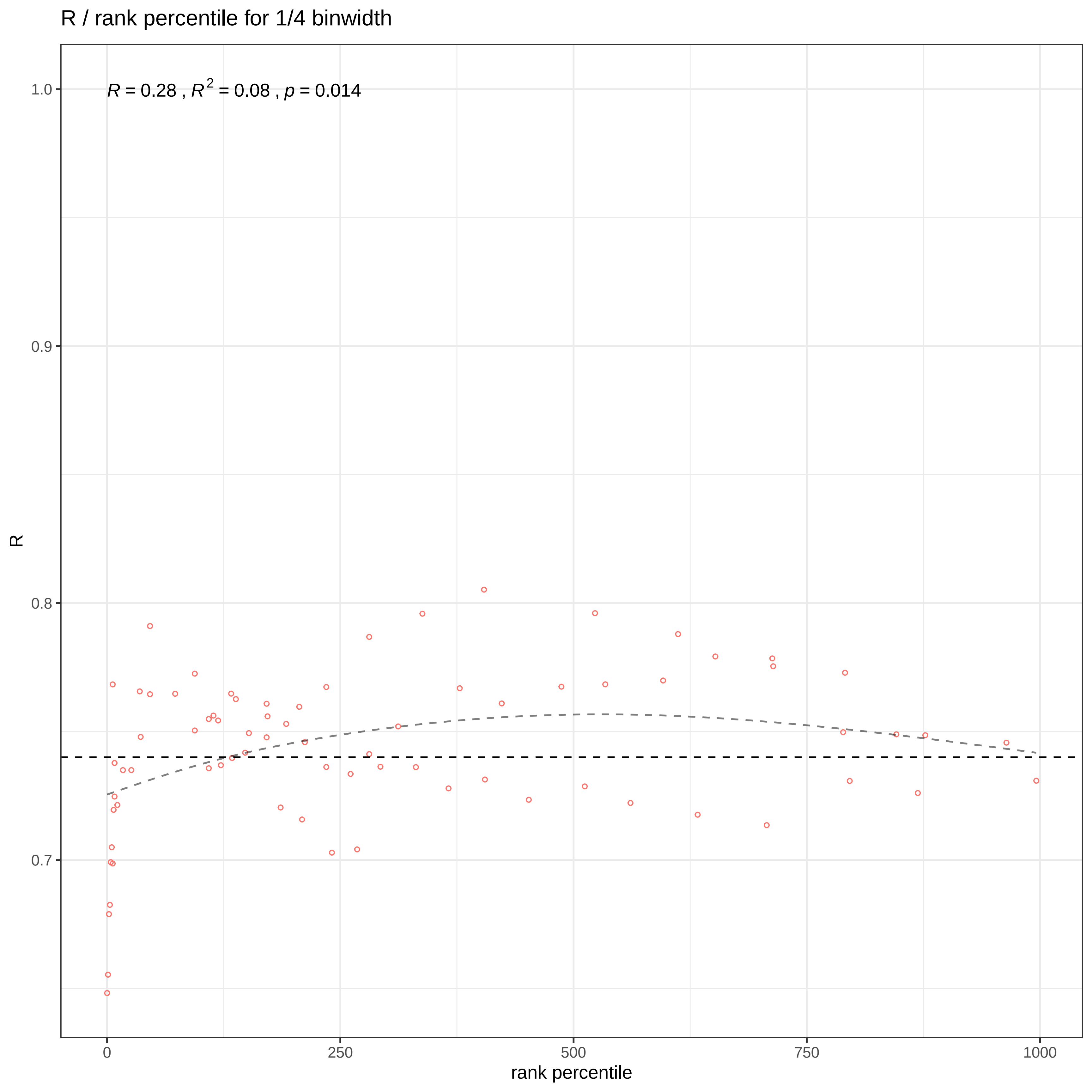
R / rank percentile for 1/4 binwidth
$R = 0.28$, $R^2 = 0.08$, $p = 0.014$
R
rank percentile
0.7
0.8
0.9
1.0
0
250
500
750
1000

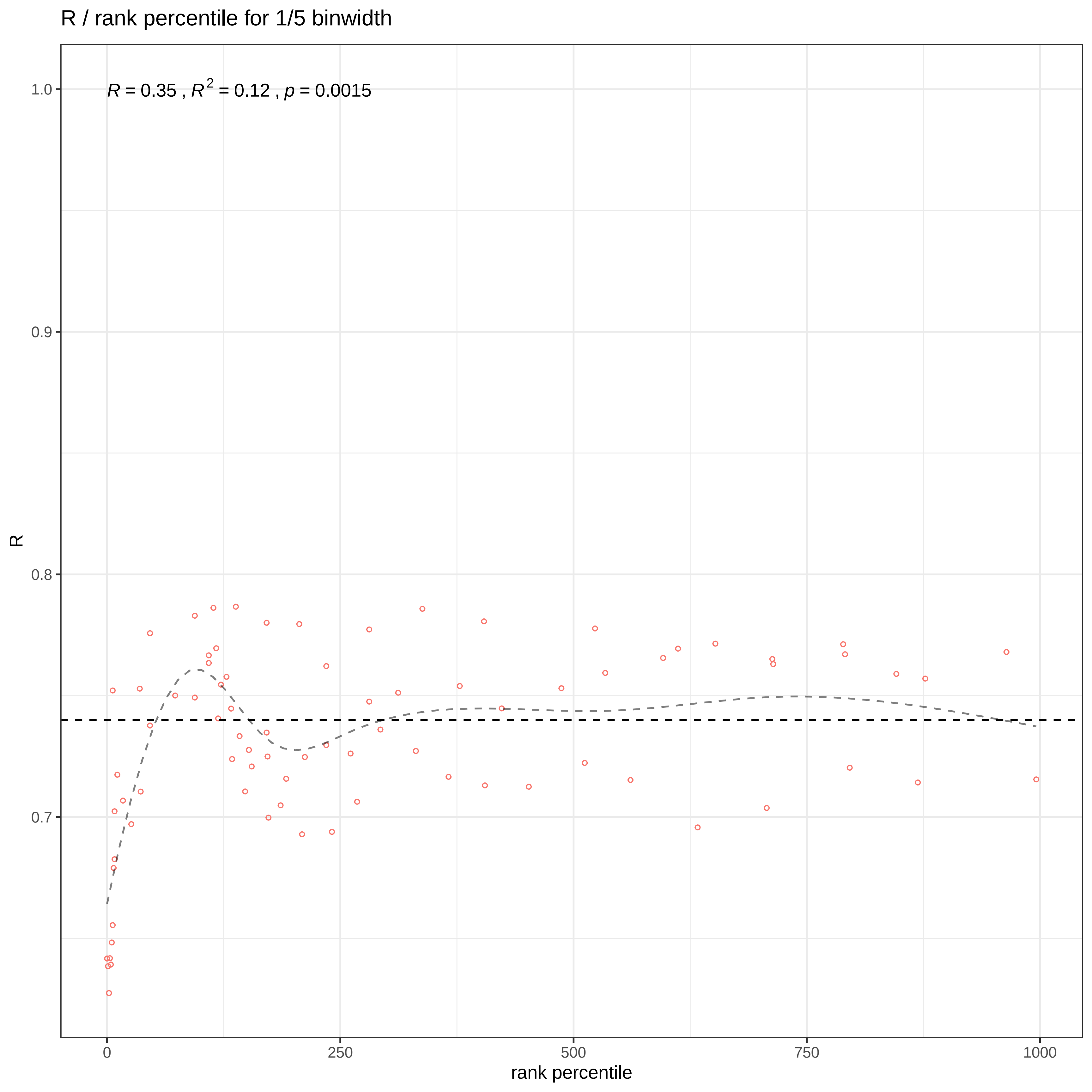
R / rank percentile for 1/5 binwidth
$R = 0.35$, $R^2 = 0.12$, $p = 0.0015$
R
rank percentile
0.7
0.8
0.9
1.0
0
250
500
750
1000

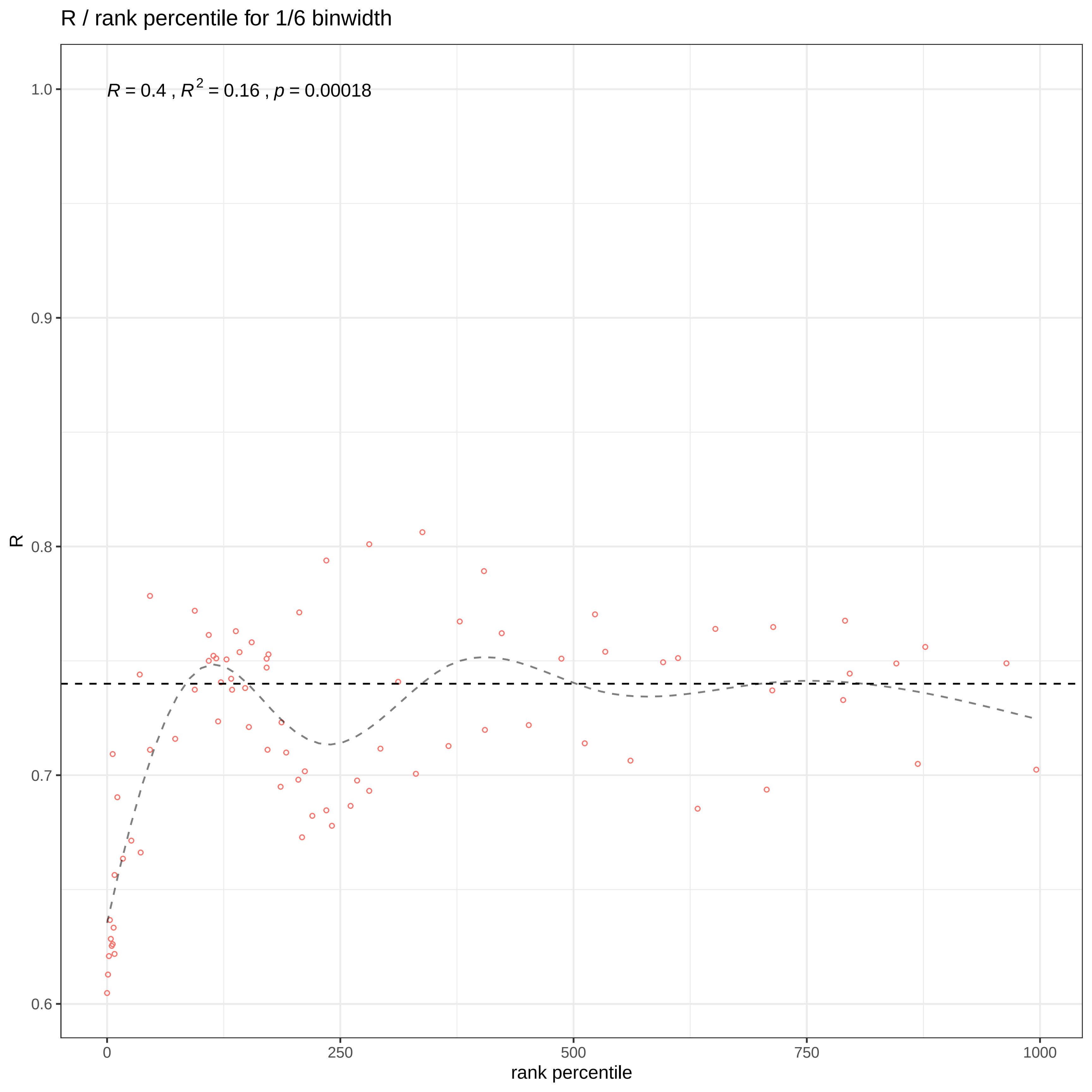
R / rank percentile for 1/6 binwidth
$R = 0.4$, $R^2 = 0.16$, $p = 0.00018$
R
rank percentile
0.6
0.7
0.8
0.9
1.0
0
250
500
750
1000

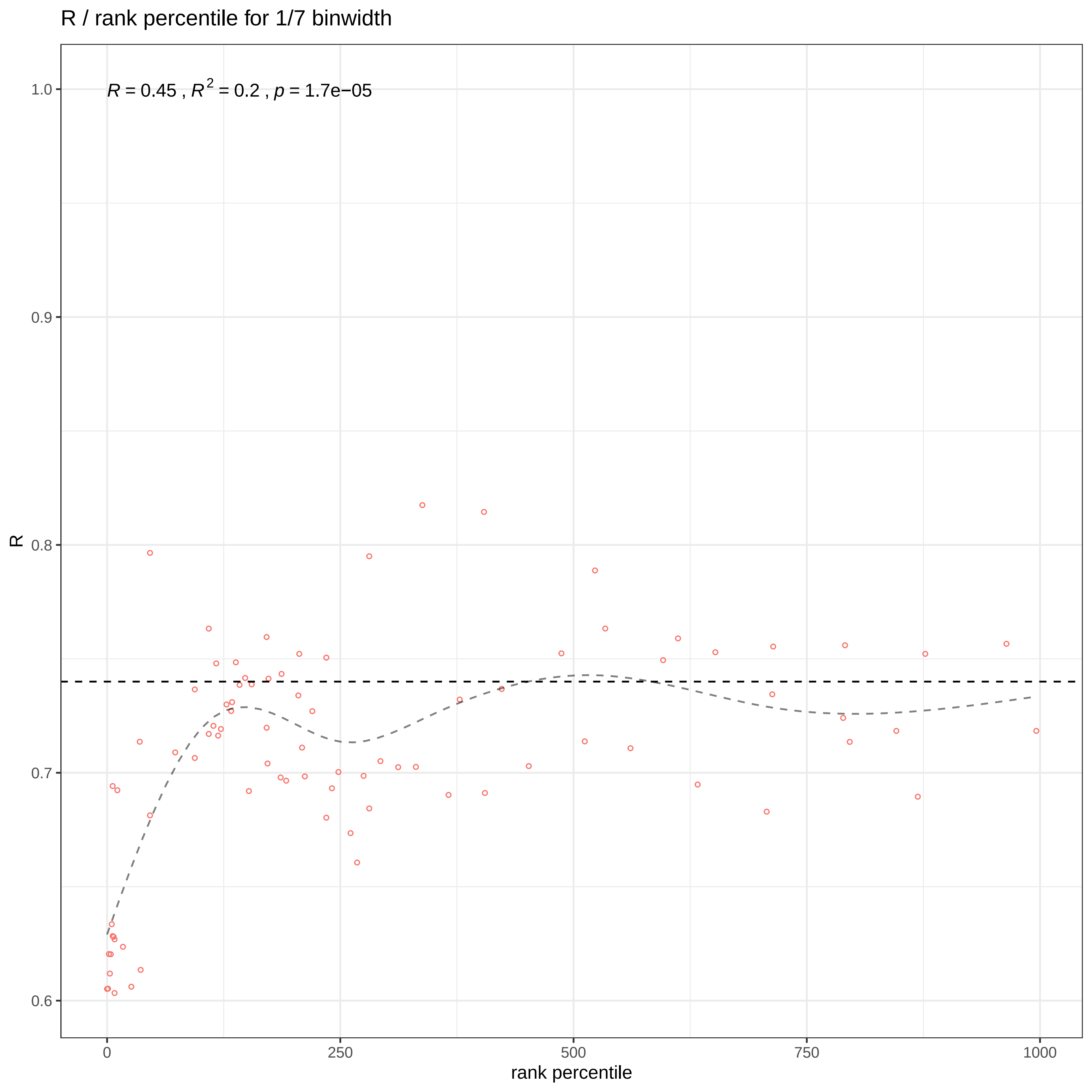
R / rank percentile for 1/7 binwidth
$R = 0.45$, $R^2 = 0.2$, $p = 1.7e{-}05$
R
rank percentile
0.6
0.7
0.8
0.9
1.0
0
250
500
750
1000

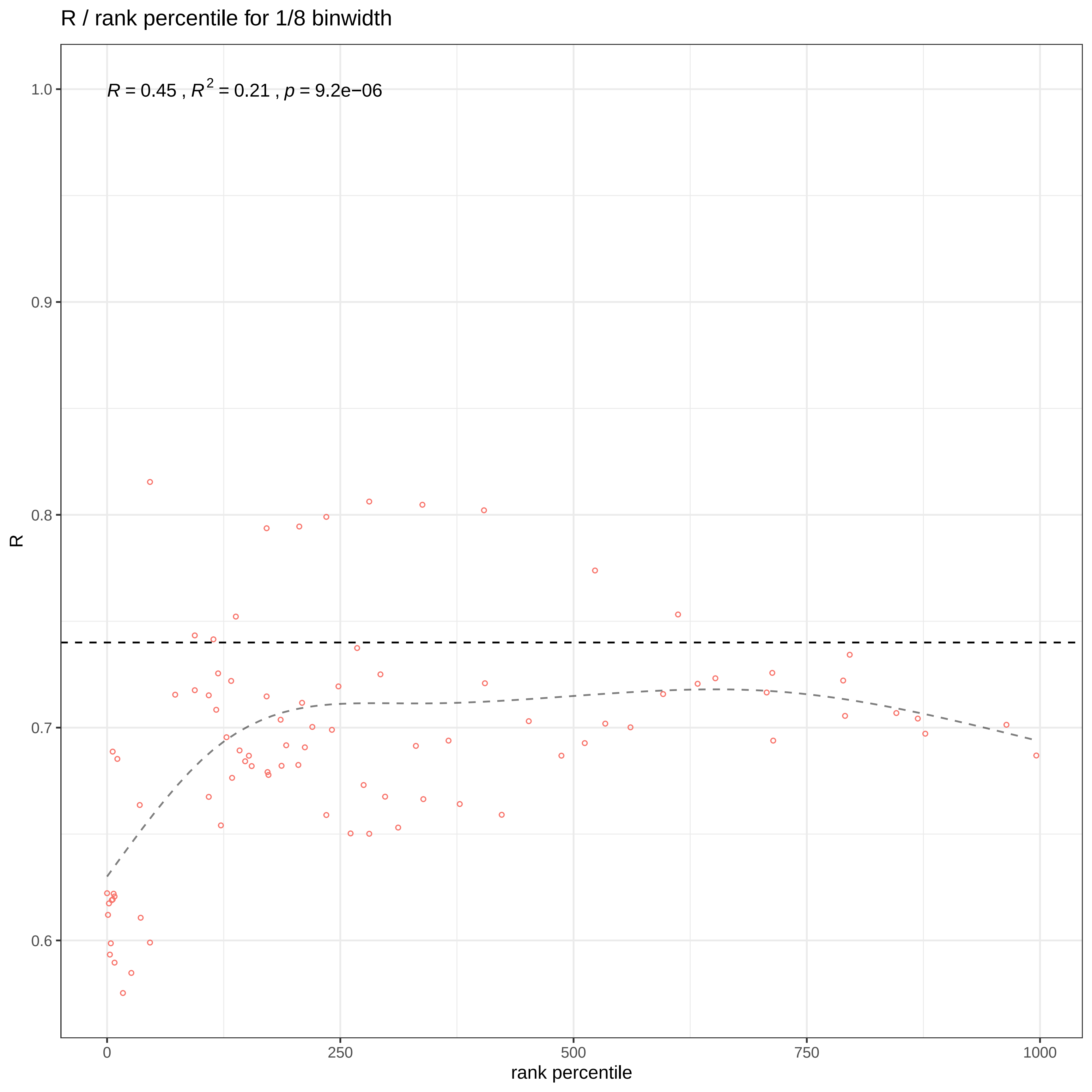
R / rank percentile for 1/8 binwidth
$R = 0.45$, $R^2 = 0.21$, $p = 9.2e{-}06$
R
rank percentile
0.6
0.7
0.8
0.9
1.0
0
250
500
750
1000

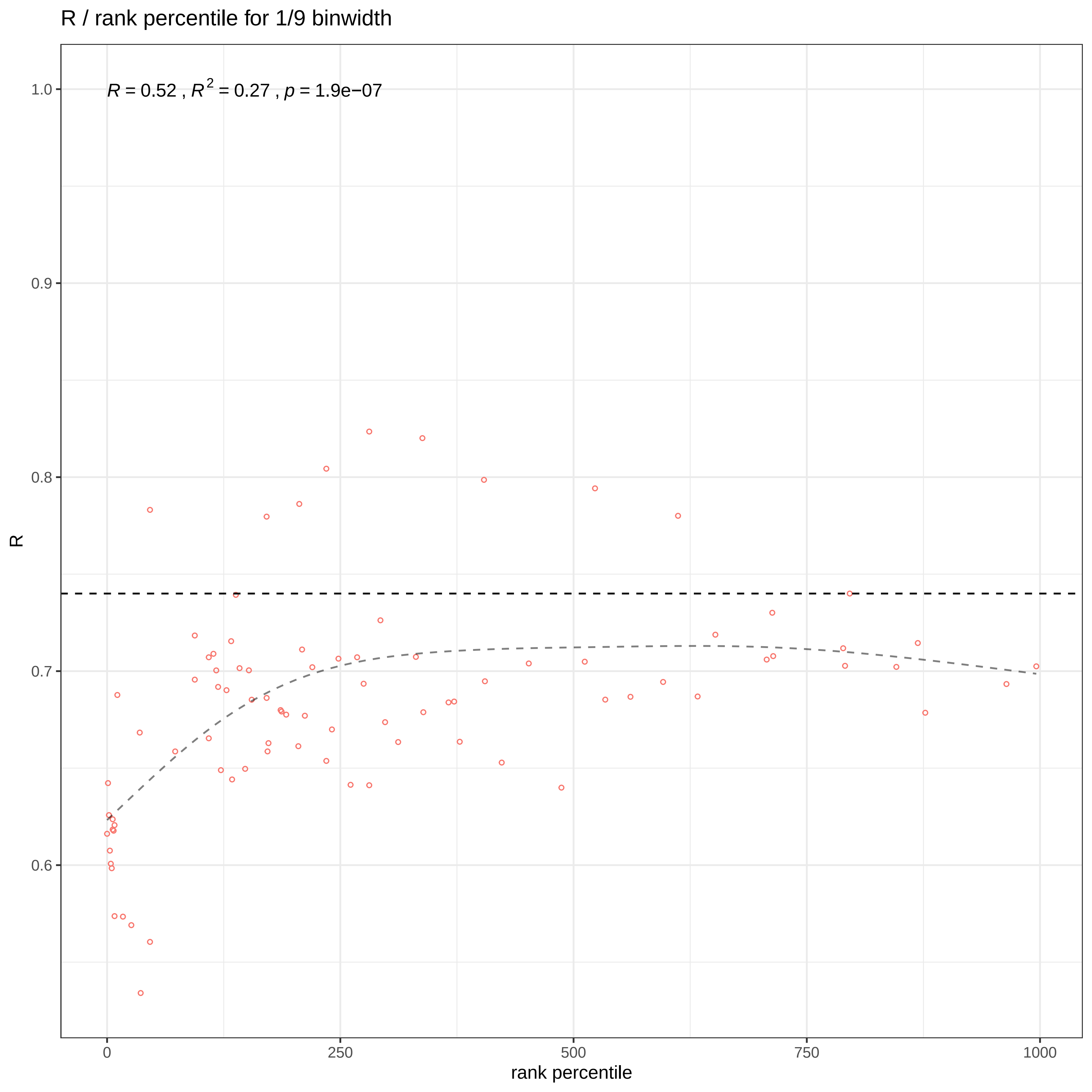
R / rank percentile for 1/9 binwidth
$R = 0.52$, $R^2 = 0.27$, $p = 1.9e{-}07$
R
rank percentile
0.6
0.7
0.8
0.9
1.0
0
250
500
750
1000
