# Supplementary material for: Genes for highly abundant proteins in Escherichia coli avoid 5’ codons that promote ribosomal initiation
Source: PLoS Comput Biol. 2023 Oct 25;19(10):e1011581. doi: 10.1371/journal.pcbi.1011581 (PMC10599525; doi:10.1371/journal.pcbi.1011581)

Fig S3. Codon usage in Essential genes. A. Codons enriched at the 5'-ends of essential genes in E. coli are also enriched at the 5'-ends of native highly expressed genes. The x axis is the is the log odds ratio for the codon being enriched at the 5'-ends of E. coli genes with high protein abundance compared to low protein abundance. The y axis is the log odds ratio for the codon being enriched at the 5'-ends of essential genes compared to non-essential genes. **B. Essential genes in *E. coli* are depleted in optimal translation initiation codons compared to non-essential genes.** The *x* axis is the log odds ratio for the codon being associated with high protein expression levels when used at the 5'-end in experimental transgenes (edIO). The *y* axis is the log odds ratio for the codon being enriched at the 5'-ends of essential genes compared to non-essential genes. For both figures, each data point is labelled as the codon it represents. **C.** As for figure A, but comparing all pairwise combinations of synonymous codons (i.e. within the same codon block: N=87). The pairwise differences are oriented such that, on the x axis, the codon with the lower value of the log odds ratio has its value subtracted from that of the higher value. The orientation is preserved for the y axis. This way no values on the x axis are negative. Each point is labelled by the oriented codon pair (first codon in the pair has the higher x-axis value, as seen in the A figure). **D.** As for figure B, but comparing all pairwise combinations of synonymous codons (i.e. within the same codon block: N=87). The pairwise differences are oriented such that, on the x axis, the codon with the lower value of the log odds ratio has its value subtracted from that of the higher value. The orientation is preserved for the y axis. This way no values on the x axis are negative. Each point is labelled by the oriented codon pair (first codon in the pair has the higher x-axis value, as seen in the A figure). For all figures, Principle Components Analysis (PCA) was used to fit an orthogonal regression line. The Pearson's correlation coefficient and p-value are provided within the figures.

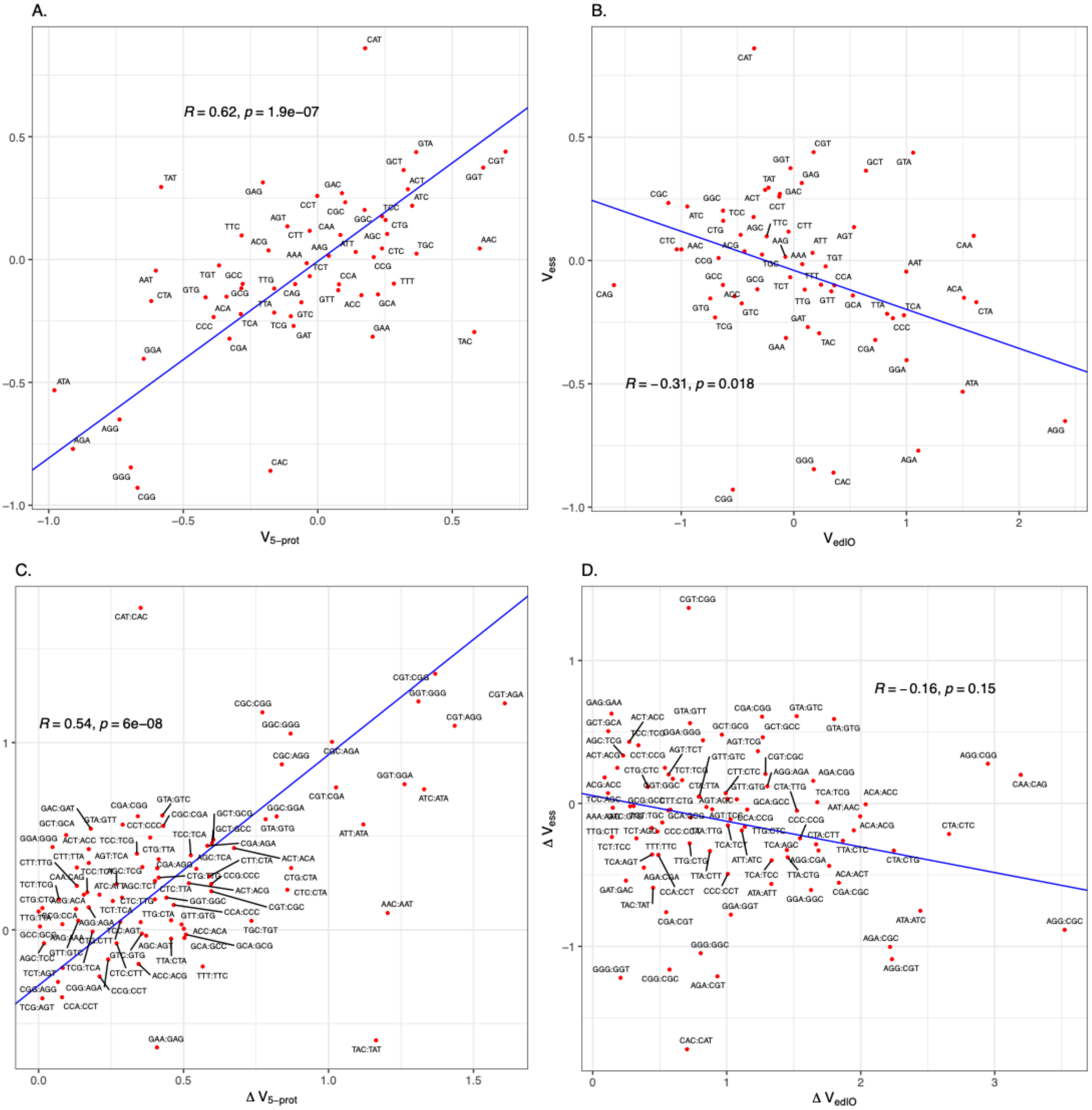

Supplement: S3 Fig — (PDF) [file pcbi.1011581.s007.pdf]
