# Supplementary material for: Genes for highly abundant proteins in Escherichia coli avoid 5’ codons that promote ribosomal initiation
Source: PLoS Comput Biol. 2023 Oct 25;19(10):e1011581. doi: 10.1371/journal.pcbi.1011581 (PMC10599525; doi:10.1371/journal.pcbi.1011581)
Supplement: Attachment — Submitted filename: Response to reviewers.pdf [file pcbi.1011581.s010.pdf]

Response to reviewers

Reviewer #1: In this manuscript, Lewin et al. computationally analyze the codon usage within the 5' region of bacterial mRNAs from indicated categories (sets). For this purpose, the authors define the "log odds ratio" to quantify the relative enrichment of a codon between two sets of mRNAs to be compared. Some interesting features on the 5' region of mRNA have been concluded from these analyses, such as codons ending with A or U promoting high protein levels, enrichment of optimal codons for translational initiation. The most intriguing finding is that, in E. coli, genes for highly abundant protein avoid 5' codons that promote translational initiation, as illustrated by the title. Overall, the results from this manuscript provide insights into the mechanism of translation in bacteria and will be useful for the design of expression vectors. The followings are some concerns I have.

In Fig. 1, the AGG codon (for arginine) shows the highest log odds ratio among all 59 codons, especially it is an exception (ending with non-A/U). This phenomenon deserves more discussion. In particular, the AGG codon is a rare codon and, as the authors mentioned (reference #85), NGG (including AGG) codons are suppressive in the 5' region of mRNA.

Response: We agree that this AGG usage is a most interesting and unexpected result. We have checked as to whether it may just be that the high logs odds ratio is owing to low numbers of the relevant amino acid in the Goodman data and to this end calculated the standard error for all log odds ratios (now added to figure 1). It isn't that. We also checked back with the Goodman data – they present an alternative metric of enrichment – and they also report AGG as the highest scoring, so it isn't an epiphenomenon of our using the more classical log odds ratio. To some degree it is possibly an artefact of the method to compare within synonymous blocks as the 4-fold block of arginine all start CG. Thus if arginine is to be used we would expect preference for AGA and AGG over CG[C/G]. This accounts for part of the effect as if we treat AGA/AGG as a two-fold block the log odds ratio is much less extreme. We also now additionally note something we hadn't noticed before – AGG also has the most negative log odds ratio in translational optimality scores (ie most under used compared to synonyms in the core of highly expressed genes). This we propose fits with Tuller's suggestion that there might be selection for translational slowing as well as 5' low stability associated with efficient initiation (even though Bentele et al's experiment results argue against this).

We have added a paragraph to the discussion addressing all these points:

Despite the above, the extremely high overall IO value of AGG (Fig 1) is an enigma worthy of further investigation. It isn't a sample size artefact, the standard error being relatively small (Fig 1). However, the value for any given codon is also dependent on the usage of the synonyms. In this regard, AGG is unusual in that it has four synonyms that are GC rich. AGGs high value may thus in part reflect a more general trend for high log odds ratios of the two AG starting codons for arginine, as opposed to the CG[G/C] ones (N.B. within the 4 fold block the A ending codon has the higher ratio). Indeed, if we define AGA and AGG as a two-fold block then the log odds ratio for AGG enrichment is 1.12, which is no longer the highest value. Nonetheless, there remains the mystery as to why AGG has a higher logs odds ratio than AGA, the codon that more obviously obeys the A/T over G/C preference. One possible clue comes from comparison of V_{TO} *and* V_{edIO} *scores. It can be no accident, we suggest, that AGG both has the highest log odds ratio for the later and the lowest for the former (S2 Fig). This would support the*

hypothesis that selection on 5' ends may not simply be mediated by selection for low stability but also for translational non-optimality, possibly owing to effects on ribosomal velocity [60] (but see [54]). Similarly, while within the 6-fold leucine block the two A ending codons (CTA, TTA) have the highest log odds in the Goodman et al data, prima facie CTA should have the lower value given its first site usage but doesn't. However, consistent with a possible effect of ribosomal speed, CTA has a lower TO score than TTA.

Also, the y axis label of this figure should be "log odds ratio".

Response: Thank you. The mistake is corrected (and we have also changed the colour scheme – see below – and added error bars).

Other minor points:
* RNA "stability" used in this manuscript is exclusively to indicate the "structural stability" of RNA. However, some readers of different backgrounds may mis-regard it as, say, the resistance of RNA to degradation. The authors should make it clear in the context.

Response: thank you for this useful point. We now write:

Note that the term RNA stability in this context, refers exclusively to energetic stability, commonly measured as Gibbs free energy, of predicted RNA secondary structures and not to RNA degradation or measures of half-life.

* p.15, line 2 from bottom: My understanding is that the histogram per se in Fig. 6 is "left skewed", though the authors would emphasize that the distribution is largely shifted to the right from zero.

Response: to avoid confusion we replace the word skewed with shifted as the point is most data sits to the right of zero

* p.16, line 8: "discrimination from the core codon content will we low." A typo: we -> be

Response: corrected

* p.19, line 10 from bottom: "Usage of the 7 codons that are positive for IO and TO would be an obvious starting position." What are the 7 codons?

Response: Added. As it happens we had already defined them in the main text but it is certainly worth restating.

Reviewer #2: Lewin and colleagues perform an in silico analysis of nucleotide sequences in general and codon sequences in particular. Independent recent studies concluded that synonymous codon modifications that reduce 5' mRNA stability result in increased protein levels. The goal of the present study is to reveal relevant details that determine the expression levels in E.coli, both of natural genes and of transgenes. Surprisingly, this study revealed that the 5' ends of native genes that specify highly abundant proteins avoid experimentally demonstrable initiation optimal codons. An interesting study, but there are concerns that

should be addressed.

Major comments:
1. Although the method employed in this study appears valid and yields interesting data regarding the codon usage in HEGs in E. coli there are some conceptual unclarities in this work that require further elaboration. As the authors describe in the introduction, various studies (Kudla et al., Goodman et al., Nieuwkoop et al., Hollerer & Jeschek) convincingly demonstrate that codon frequency and/or tRNA abundance do not directly reflect the corresponding protein level, but rather that this appears to be better predicted by the potential mRNA folding of the region around the translation start at the mRNA transcript, including the 5'UTR and the 5'CDS. Therefore, one could conclude that the 'optimal initiation codons' would be context-dependent, and are dependent the sequence of the 5'CDS and 5'UTR. With this in mind, could the authors explain why they still decided to use codon usage as a sole measure for translation efficiency?

Response: There are two issues here: 1) is our codon based metric conditional on the UTR employed and 2) why do we focus on codons?

Regarding the first, this is an important caveat to our results that we only partially addressed before. It is indeed the case that in principle the IO score for any given codon at the 5' end might be contingent on exact nature of the 5' UTR employed. In principle it could also be contingent on the 3' or specific local sequence. We have performed some further analyses to consider this issue as well as discuss the problem more generally. First, we now also re-examine the correlation between the Gold-standard and E. coli's native gene 5' enrichment (compared to core) as the HEGs will be diminishing this correlation. We find that controlling for this the correlation can go over r=0.8, which suggests the Gold standard is capturing something well. Second, if context matters, we expect to see that the log odds vector might be conditional on position in the 5' N terminus as well. To this end we test for internal consistency between the 59 element vectors for positions 2-11 in the Goodman data. We find it is "excellent" (alpha =0.95!). We have added discussion as to whether our Gold standard is the gold standard that we presume it to be highlighting the possible context dependency. We argue that it is the best currently available and adequate for what we wish to claim:

Perhaps the most important caveat to our analysis concerns the assumption that the Goodman et al. data provide a Gold standard for estimation of initiation optimality scores (initiation optimality being broadly defined). The values we have derived will no doubt vary with the thresholds for the most abundant and least abundant classes (we employ top and bottom 25% by Prot.FCC). Perhaps more importantly, as above, our metric does not factor in any aspects of context. This could mean codon-by-position effects (above) or, possibly more importantly, the impact of the 5' UTR employed. Indeed, if the mechanism of action is mediated by RNA folding and stability effects, we might expect that the 5' UTR composition would be important in determining the initiation optimality scores for the 5' codons. To determine this one would need a more extensive experiment in which amino acids, codons and 5'UTR are all extensively randomised. We are unaware of any such experiment.

While in principle a serious issue, we have reason to suppose that our IO scores are, however, fairly robust. First, we recover a trend for high A/U content promoting expression as do all prior experiments [37, 40-42]. Second, this A/U preference is seen in cross-species analyses [41]. Third, the log odds enrichment scores appear to be highly consistent between all N terminal positions (see above). Were context to be especially influential we would have expected that log odds ratios would show more position context dependency. Fourth, in E. coli there is a robust correlation (r=0.74) between the Gold standard vector and 5' enrichment vector (Figure 5). As this is in part diminished by inclusion of the highly expressed genes, we can also see what happens when we break the data in to

bins by expression level, derive a 5' enrichment vector for each bin and consider how these vectors then correlate with our Gold standard vector. Doing this we find that the correlation can exceed r=0.8, the correlation being weakest for both the most highly expressed (under conflicting selection) and the lowest expressed (probably under the weakest selection) native genes (S5 Fig). That the Gold standard vector also predicts 5' enrichment in many bacteria (Fig 6) similarly suggests that it isn't too misleading. We consider it viable to still consider it the present Gold standard (best current estimate), but whether it is definitive requires further experimentation. We suggest that it is robust enough both to substantiate our claim that HEGs avoid IO codons (Fig 2) and of utility for the design of 5' ends of transgenes.

To the second point as to why we focus on codon usage, this comes back to the motivation for the paper. Why we are interested in defining scores for each codon also runs for the same logic as for defining CAI for every codon: it could possibly be used as a really simple metric to estimate expression levels (we now think not given our results) for non-model organisms and could most particularly be employed to design transgenes just as CAI scores have historically informed codon choice. Algorithmically this is how many transgene design programs work – they take each amino acid and for each derive a codon to use. In essence, what we are attempting is to do for translation initiation – very broadly defined – what CAI does for translational optimality. Both treat the underlying mechanism as a partially obscured black box and both use surrogate metrics. We hope the referees can appreciate our motivation and thus the choices made.

Perhaps we could bring the referee's attention to the key paragraph in the introduction:

We here are not so much concerned with the mechanism by which the 5' codon effects manifest alterations in protein levels, nor are we concerned with determining which parameters best predict protein expression levels, this being well resolved [42]. Rather we seek to ask whether it is possible to determine from a purely in silico analysis the set of codons that are initiation optimal for any given bacterial species (or more generally IO scores for each codon), just as we routinely attempt to infer codon adaptation indexes [63].

We also now write:

That TO codons do not majorly affect protein level while IO ones do (see above), provides an immediate rationale for finding a simple method to determine IO codons of any given species. This in part motivates our focus on codon composition (as opposed to, for example general 5' stability) as transgene design algorithms commonly employ codon-level metrics to determine which synonymous site to employ within the transgene, sometimes in a gene position specific manner (e.g. [65-67]). Second, while CAI provides a useful surrogate for expression level of a gene in some species [8, 36], understanding IO scores could augment such a metric providing a better proxy of protein abundance, potentially applicable also in species in which there is no evidence for translational selection [36] and no (hard to obtain) protein level measures. A simple metric of mean 5' IO score may then be useful. This again informs our choice to define codon specific IO measures.

Would the RNA stability not be a better indicator for expression level?

Response: Again this comes back to what the aim of the paper is. As we said, the function of the paper is not to derive the relative contributions of all possible parameters. This we think the referees, along with Cambray et al have done well. As we noted in the discussion, if one really wants to predict expression level for any given native gene then a complex machine learning approach is almost certainly preferable. Our much simpler point is that IO scores are unexpectedly not good predictors. It would have been lovely if a simple IO metric was a

really good predictor of native expression, it being derived from a transgene experiment to consider just this effect. But they are not.

The above addition addresses the RNA stability issue specifically. On a more pragmatic level, even if our aim were to determine predictors of protein level (which it isn't), as we intimated, we are very reluctant to employ RNA stability measures to deduce expression levels. We can put more flesh on these reservations. First, we don't think any RNA folding package is up to the job yet for many reasons (they ignore proteins on RNA, they don't get benchmarked observations right etc). Second, it isn't clear that stability minimizing is the relevant metric. When we look in the AT richest bacteria, the 5' ends actually have higher GC3 content than the gene body suggesting low but not minimal stability may be optimal. Third, it isn't clear that stability, even if we could accurately measure it, would be the key variable – it may be a proxy to non-hiding of SD sequence as we discussed. Fourth, as we now show the AGG effect may be explained by it having the lowest V_{TO} (CAI) score supporting the hypothesis that expression level isn't simply mediated by RNA stability. However all of this is moot as our aim is not provide a statistical model to predict all native gene protein abundances (although this would be an interesting challenge).

The Golden standard dataset reports data on 5' RNA stability for the analyzed constructs. Could a similar analysis be done for the selected HEG dataset of this study and could information on the folding energies of the studied HEGs reveal additional insight in the 'choice' of codon usage of HEGs?

Response: we have looked at Gibbs free energy of the 5' ends of native genes, as predicted by the Vienna package (RNAfold) and see no correlation between protein level and predicted stability. The data is very noisy with extensive variation in free energy in all expression level bins. We report this but don't want to make anything of it as net expression level is likely to be a combination of so many factors. Additionally, we have little faith in estimates of stability both because these are short fragments and given the known inaccuracy of folding programs. The analysis may have relevance when considering Goodman's transgenes as all other parameters are then controlled. In short, we don't think we can – or should – derive anything much from this but are happy to report it at the referees' request.

2. An important question is whether the Golden standard dataset the right reference for this approach? Since the Golden standard dataset is a result of a library where 5'UTRs and 5'CDSs were (randomly) combined, is it not possible that Goodman et al. selected for combinations of 5'UTRs and 5'CDSs with A/T-rich, rare codons resulting in e.g. low folding energies? This is very useful information for transgene expression, but do the sequences in the library of Goodman et al. also reflect the in vivo conditions of HEGs in E. coli and does that allow for direct comparison of the correlation between expression level and codon usage?

Response: It was concerns such as the above that motivated our choice of metric, namely the log odds ratio. A key point about this metric is that it should be independent of any skew in Goodman et al's choice of amino acids and codons at 5' ends as it asks for any given codon its usage in the highly expressed compared to the lowly expressed transgenes, also making allowance for the usage of the synonymous codons. Thus over or under use of certain codons

in the constructs *en mass* will not affect the log odds ratio as it internally controls for such under or over usage.

This we think is also why the Gold standard predicts 5’ codon usage in E. coli overall really rather well (just not, unexpectedly for the most highly expressed genes). As we now note if we consider tranches of the data the correlation between the 5’ core enrichment and the gold standard vector is over r =0.8. In our experience in biology correlates of this strength are almost unheard of. And note that these are perfectly independent data sets!

We now clarify stating:

Note that this method excludes any effects of skewed amino acid or codon usage in the constructs and controls for transcript level differences.

Do the expression levels achieved by Goodman et al. match the expression levels of the studied HEGs, or are they higher/lower?

Response: We had a look at the original Goodman supplementary data file and it has a column called Count.Prot which we assume is comparable to the absolute protein data we took from PaxDB – we can’t be quite sure as Goodman’s supplementary data is not especially transparent (we originally wrote to them for clarification but received no response). If this is the case, then they both seem to fall within similar ranges - highest value for Count.Prot is ~75,000 while the highest for native E. coli protein abundance is ~82,000. The mean is however a bit higher in the Goodman data (~6800 v 1000) but they also note that they couldn’t get accurate measurements when the level went very low.

Goodman et al. used two different promoters which allows for normalization on transcript level between 2 datasets, while HEGs will each be under control of their own promoters. Could there also be an additional effect of transcript level on the expression level of the HEGs? Please elaborate.

Response: We did touch on this previously noting that, given that HEGs appear not to have strong initiation, different native protein abundances most likely reflect other factors such as differential promoter usage. We now expand this:

Furthermore, even within E. coli, we cannot go from the scores in the V_{edIO} *to derive the expected protein abundance of native genes, presumably because promoter, RNA half-life or protein half-life differences are key to explaining intragenomic variation in protein levels.*

Also note that the dataset used by Goodman et al. did not employ 147 different target genes (as the authors mention in the Materials and Methods (page 22, first paragraph)), but the 11 first amino acids of 137 endogenous essential E. coli genes cloned at the 5’end of an sfGFP reporter gene. The statement that this dataset is superior as it employed 147 target genes rather than just one target gene in other studies therefore is misleading and should be rephrased.

Response: Thank you for spotting this error: the 147 was a typo – indeed 137. As regards the utility of this dataset, as they employed many different 5’ ends in terms of amino acid content

all codons were utilized so we can obtain data on all codons – we clarify wording and explain better the nature of the constructs. We now write:

To assess the experimentally derived initiation degree of optimality (edIO) of each codon, a log odds ratio was calculated describing its enrichment at the 5'-ends of highly expressed experimental transgenes compared to lowly expressed transgenes, allowing for variation in transcript abundance. The data comes from a large-scale transgene experiment in E. coli that measured the expression levels of 14,234 constructs with different combinations of promoters, ribosomal binding site (RBS) and N-terminal sequence [38] fused to an sfGFP reporter, the level of protein derived being compared to an mCherry reporter under a constitutive promoter on the same plasmid. The amino acid content of the N terminal ends (first 11 codons) was taken from 137 different native E. coli essential genes. Because of the employment of multiple 5' ends differing not only in codon usage but also amino content, this data set is unique in that we can obtain log odds ratios for all 59 codons that have at least one synonym. The same is not true for any alternative data set as they preserve the 5' amino acid content of the construct.

For each N terminus, promotor, RBS combination Goodman et al [38] generated 13 different constructs differing only in synonymous site content in the first 11 codons. One of the 13 was designed to employ translationally optimal codons, one translationally non-optimal codons and the others varied in predicted RNA stability. For each group of 13 they determined fold enrichment of all constructs (compared with the mCherry control) against the mean of the 13, this being their metric Prot.FCC. This is comparable between groups of 13 [38].

3. The introduction is rather lengthy and contains a lot of 'woolly' language, convoluted sentences and repetition. Overall, this makes it difficult to read. Please make the introduction more concise. Specifically, pages 3 and 4 all describe the single concept of codon usage not relating to protein expression level which could be significantly condensed. Also, the rationale of the study (pages 6 and 7) repeats many concepts previously introduced.

Response: The introduction has been tightened up.

4. In the introduction (page 5, last paragraph), the authors provide a description of IO and TO codons, essential for further understanding of the paper, but the given definition or distinction between the two is fuzzy and sometimes contradicting. What exactly constitutes either a TO or IO codon, is there a threshold or definition? Could A/T-rich TO codons also be IO codons? Please clarify a (concrete) distinction between the two.

Response: We agree that there is ambiguity here as there is in much of the codon usage literature. We think there are two sources of ambiguity. The first is that "optimal" codons are defined both by a mechanism and by a proxy measure. TO codons are those that match the most abundant tRNAs (mechanism) but measured as enrichment in highly expressed genes (proxy definition, CAI or our V_{TO}). Likewise, IO codons we suppose to mediate initiation probably via stability effects at least to some degree, but we measure them via the Goodman data, enrichment in the 5' ends of transgenes associated with high protein abundance given transcript abundance compared to alternative constructs.

The second ambiguity is that we talk of "optimal" and "non-optimal" codons in a digital manner, but in practice we have a continuous variable as the metric (CAI = V_{TO}, V_{edIO}). There is thus no cut-off, excepting if one wants to consider a log odds ratio > 0 as in some manner optimal.

We now clarify the digital/continuous issue early on:

Notice that codon optimality can be both considered as discontinuous concept whereby for each synonymous block there is one optimal codon all others being non-optimal or a continuous variable, each codon being granted a score dependent on its degree of enrichment in HEGs.

We are also more careful to talk of codons with high CAI/TO/IO scores. But the whole field talks of optimal codons in analog and digital modes interchangeably with the same inevitable ambiguities. We hope our usage isn't too confusing now. Formal definitions of both V_{TO} and V_{edIO} are in the methods.

As regards the question as to whether TO and IO codons can be the same we previously highlighted the 7 codons that have positive scores on both measures (this is one advantage of using a log odds ratio as it presents zero as an obvious cut off).

5. In the Results (figures 2 – 5, and page 9), the rationale behind the data representation in panel B is not entirely clear. It is clear how the data in the figure is obtained, but it is not explained what new information this representation of data brings, especially as it not only compares the expression between IO and TO-codons, but also includes comparison within 'non-IO' vs. 'non-IO' and 'non-TO' vs 'non-TO' codons with each other. Does this not introduce a lot of noise? Please clarify the rationale behind this data representation in more detail.

Response: Here we are concerned with a statistical nicety that is considered more important in other fields. We think that there is a possible non-independence problem here. Indeed, for a two-fold codon set if the log odds ratio of one is *x*, then the other must be exactly *-x*. Counting these as two independent data points we think could be statistically problematic potentially inflating significance – is this one data point or two? As with the method of phylogenetic contrasts, where you consider the difference between two species as the metric, so too we think here, to be cautious, we should present the data in a manner that removed the non-independence between data points by considering the difference between synonymous codons (for which there is precedence) treating the difference as one data point. It also allows us to make strong statements about comparisons between synonyms that would not otherwise be possible such as:
The same trend is seen when comparing between synonymous codons within a synonymous codon block, the codon that more enables higher expression in a transgene is the one avoided in the 5' end of the genes for the most abundant proteins (r= -0.3, P=0.005, Fig 2B).

We now add a section to the methods to explain the rationale:

Comparison between vectors.
We employ two methods to compare any two vectors of log odds ratios. In the first we consider a Pearson product moment correlation between the two vectors (to check for outlier effects we also employ Spearman's rank correlation where stated). This may however have a statistical problem given the nature of the log odds ratio calculation, namely that the values for any codon are not independent of the values for the synonyms. Indeed, for a two-fold codon set if the log odds ratio of one is x, then the other must be exactly -x. As with phylogenetic nonindependence, there may be a problem of inflation of sample size by considering both x and -x. To be cautious that results are not artefacts of this non-independence, following [98] we consider the difference between log odds ratios between all pairs of synonymous codons, each difference being a data point. In the case of two-fold codons for

example, we employ one difference value as one data point rather than two (x and -x). For each comparison we present the correlation of the raw values (as plot A) and the correlation of the pairwise difference plot (as plot B).

Minor comments:

6. Page 2 - line 4: "concept of the translationally "optimal" codon": The definition is not clear, is this the most abundant codon or the codons present in HEGs? The next sentence suggests this is not the same.

Response: we only ever employ enrichment in HEGs. We have reworded to remove any ambiguity.

7. Page 4 - Paragraph 2: "While this accords with […] processing be too slow" This long sentence is very convoluted and it is unclear what the authors mean, please rephrase.

Response: this whole section has been rewritten to be tighter.

8. Page 4 - last paragraph, first sentence: Misses a word (assuming the effect of?)

Response: Rewritten to be more exact:

Assuming that CAI does not well predict transgene protein levels…

9. Page 5 - paragraph 1: Typos: Hypothesied (Hypothesized), Nieukoop (Nieuwkoop)

Response: corrected

10. Page 5 - paragraph 2: The related ramp hypothesis is not only dependent on 5' folding energy, but the cited paper shows it is a combination of folding energy, charge and codon bias of the sequence, indicating there is a (partial) CAI effect on the 5'CDS. How would this relate to the here presented findings?

Response: we previously addressed this issue further down in the text but now add it immediately after. Specifically, Bentele et al. show that Tuller's claim for selection for codon non-optimality isn't obviously correct. The problem is that non-translationally optimal codons tend to be AT rich, so selection for AU richness will de facto result in increased non-optimal translational codon usage. The key question then is what happens when the optimal translationally codon is AU rich? If Tuller is right that should decrease expression. Bentele et al show that in this context A/U causes high expression hence translational optimality per se is not causative, contra the correlation based proposal of Tuller. We now write:

There are multiple, not mutually exclusive, mechanisms by which 5' mRNA stability might matter, all of which suggest that low stability of the 5' end affects translational initiation broadly defined [38, 39, 50], i.e. meaning the chance that the RNA is bound by the ribosome, the ribosome finds the start codon or the translation is effectively progressed without early RNA degradation. As the ribosome has helicase activity [58], it is likely that strong 5' RNA folding is a barrier to such initiation. Strong RNA folding could also force inaccessibility of the upstream Shine-Delgarno (SD) sequence [59] that in turn affects mRNA and protein levels, this explaining why 3' mutations disrupt translation if interacting with the SD sequence and why RNA structures tend to avoid SD interactions [59]. RNA stability may additionally mediate susceptibility of the mRNA to degradation, determined by the extent

to which the early mRNA is uncovered by ribosomes [60]. The ramp hypothesis proposes that any RNA stability effects have their benefit through modulating ribosome velocity and efficient queuing [61]. The related suggestion that codon composition also modulates protein level by slowing ribosomes by matching rare tRNAs [61-63] is more controversial [55]. Indeed, when synonymous sites of translationally optimal codons are AT rich, usage of the AT rich synonymous sites still increase protein levels [55]. If generally true, 5' enrichment of non-optimal codons [61] is then a necessary consequence of selection for AT richness per se, not for codon non-translational optimality as such.

This being said, we now note that AGG is really exceptional in having the highest log odds ratio for initiation and the lowest for translational optimality, consistent with Tuller's suggestion (see response to referee 1).

We didn't previously go into the positive charge effect as it is irrelevant in this context as our metric compares between synonymous codons at the same N terminal end, thus removing amino acid content from the equation. Incidentally, we have also elsewhere shown that the positive charge effect evoked by Tuller is an artefact of transmembrane domains tending to be at protein terminal domains (DOI: 10.1093/molbev/mst169). We clarify:

Note that if there were amino acid level effects on protein abundance, such as an effect of positively charged amino acids at N termini [61] (but see [100]), this effect is irrelevant in the current context as we only compare between constructs with the same N terminal region.

11. Page 6 - last paragraph: The authors state that the presented method "permits us to understand the general utility of employing highly expressed genes as the end point of a monotonic continuum in determining the direction of selection." The description is vague, how do the given examples of this relate to optimality of initiation codons?

Response: these examples don't – they are to illustrate that in many contexts people have employed the low-high continuum to infer the direction of that which is favoured by selection. We are asking whether the same works when trying to derive IO scores – we find it doesn't making a potentially important counter-factual. We have rewritten to clarify.

12. Results, first paragraph: The study describes an analysis performed on the '5' end of mRNA' of the Goodman dataset and HEGs. Could the authors specify (here, and in M&M) which cut-off value for the 5' end of the transcripts was used in the analysis (how many base pairs, codons, constitutes the 5'end), is it the same length of 11 amino acids as Goodman et al. used?

Response: this was already defined in the methods (first 11 codons)

13. Figure 1: The used color scheme is not color-blind friendly and indistinguishable in black & white. Since colors are referred to in the text, please use a colorblind friendly color scale (e.g. the viridis color scale for ggplot in R).

Response: there is an irony here as LDH is colour blind and chose these colours as he can see them. Naturally we are happy to take the scale employed. The new figure has been tested on LDH and one other colour blind person (both gave it a thumbs up).

14. Please be consistent in referring to supplementary data (Supplementary Fig, S Fig, Fig S).

Response: corrected

Reviewer #3: Lewin, Daniels and Hurst present an interesting concept of codon optimization for ribosomal initiation. Authors show that initiation optimal codons are different from translationally optimal codons with little or no overlap with each other. The findings are interesting and convey a clear message. However, the following concerns/questions should be answered prior to the publication.

(1) Translationally optimal codons are associated with the transcript with high translation efficiency. However, that is not the case with initiation optimal codons. Their presence perhaps slows down the initiation rate as they are mostly found in the genes with low protein abundance. Therefore, this one to one correspondence does not exist. Therefore, I am not sure if one should use the phrase initiation optimal codons.

Response: We think we may have misled the reviewer. Our IO codons are defined by high expression in transgenes that modify just the 5' end and, we assume, are associated with high initiation rates as the metric allows for transcript level differences (as we now clarify). The oddity is that native HEGs don't employ high IO codons while other native genes do and presumably employ low RNA stability and/or high transcriptional initiation rates to enable high dosage.

(2) I believe that due to the unavailability of initiation rate, authors use protein abundance as a proxy of translation initiation rate. If the initiation rates are not available then I would recommend using mRNA copy number/protein copy number which is a better estimate of translation initiation rate.

Response: As above, to derive the initiation rates for the transgene data we do not employ protein abundance. The metric employed is Prot.FCC which controls for transcriptional level. It is in effect a measure of protein level compared to constructs with the same promoter and ribosomal binding site, controlling for differences in transcriptional level between otherwise identical constructs. We now clarify further.

(3) Authors find A/T ending codons promote high protein levels in E. coli. I think this result can be explained by low stability of mRNA structure near the start codon. Or there is something new here which I am missing.

Response: We don't think you are missing anything. We don't think this result is novel and is very expected given RNA effects – Goodman et al indeed report this result – high A/U associated with low stability as we explain in the introduction.

(4) For the clarity of the readers, authors should discuss what they mean by the noise associated with the codon and how it is low at a lower initiation rate.

Response: we now more formally define noise and add more discussion of the relationship between low initiation and low noise. The low initiation - low noise effect is captured both in simulation and in experimental data. We would prefer to point the reader to these simulations than attempt to verbally explain the result, not least because, as we point out, others claim in some parameter space the result may not hold:

Additionally, selection on native highly abundant proteins is not simply to mediate abundance but is also to enable low noise [80-82], noise being commonly defined as the ratio of the between-cell standard deviation in abundance divided by the between-cell mean abundance (i.e. the coefficient of variation). For native genes, those more dosage sensitive are expected to be both more abundant and have lower noise, both devices to prevent dose stochastically reducing too far [80-82]. Consistent with reports that more ribosomes per transcript is associated with increased abundance-corrected noise [83] (but see also [84]), low noise is presumed to commonly be the result of having few ribosomes per transcript [85]. This in turn was evoked to explain why in bacteria many key genes have conserved inefficient ribosomal binding sites [83]. If true, then there could be selection on dose sensitive genes to have high transcript levels but low initiation rates. Such an effect would also be closely coupled with the possibility that low initiation also enables greater efficiency of ribosomal usage [86]. Both noise and efficiency may then select for low initiation rates for key genes. To examine this, we consider the correlation between 5' codons associated with low noise (directly measured) and codon preferences for the native highly abundant proteins. In addition, essential genes are, by definition, the most dose sensitive of genes as, when dose is zero fitness is likewise zero [80-82]. We thus also expect essential genes to be enriched at 5' ends (compared to non-essential genes) for codons associated with weak initiation.

(5) Authors find that the transcripts that code for highly expressed proteins tend to have low initiation rate.

Response: to clarify that it isn't quite what we claim – we claim they unexpectedly have codons that are associated with low initiation rates as defined by transgene data.

Then, authors rationalize this observation by quoting previously published results that low initiation rate minimizes the noise. Another benefit of low initiation rate is that it minimizes the ribosome consumption by allowing a ribosome to produce proteins more efficiently (PMID: 37007710). Authors should discuss this additional benefit of low initiation rate in transcripts that code for highly expressed transcripts.

Response: we must apologize for this oversight – we simply were not aware of this paper. We now cite it (as above) and flag this in the abstract. We think both this model and low noise model make the same prediction (so present them as one), namely selection for lower initiation to control noise or efficiency or both. Thank you for drawing this to our attention.

other comments:
(1) page 3, "a closely related approach employs codon ... employed overall". I think this is the codon harmonization approach which is employed to enhance the production of natively folded protein. This approach is used for proteins that tend to misfold. I don't think codon harmonization is used to enhance protein production.

Response: we have gone back over the literature to see if this is the case. From our reading, usage of the observed codon frequencies is used in several contexts. We have rewritten as our original statement was probably too strong.

(2) correlation in figure 2 is statistically significant. I would recommend them testing the correlation and p-value after removing one or two extreme points.

Response: we agree that Pearson product moment can be sensitive to outliers. However, the usual control for outlier effects in correlation is not to remove data as one cannot then be certain that effects are owing to outliers as opposed to reduced sample size. For correlations, the preferred method is to consider rank ordered data as extreme absolute values are them not influential. We now report that both are significant on spearman rank correlation (in fact for Fig 2B the effect is slightly stronger and more significant).

(3) Authors may consider shifting materials and methods before the results section.

Response: we believe Plos Comp Biol's house style is as we have it.

(4) Introduction section is too long. It can be shortened.

Response: we agree and have attempted to shorten and tighten up.
