# Supplementary material for: Genes for highly abundant proteins in Escherichia coli avoid 5’ codons that promote ribosomal initiation
Source: PLoS Comput Biol. 2023 Oct 25;19(10):e1011581. doi: 10.1371/journal.pcbi.1011581 (PMC10599525; doi:10.1371/journal.pcbi.1011581)
Supplement: Attachment — Submitted filename: Response to reviewers.docx [file pcbi.1011581.s011.docx]

Response to reviewers
Reviewer #1: My previous comments and questions have been adequately reponded and answered by the authors. I do not have further questions.

Thank you

Reviewer #3: In the revised version, authors used the term high efficiency in the abstract. I think the authors meant the efficiency of ribosome usage. It should be clarified otherwise it gives the impression of translation efficiency which is different from the efficiency of ribosome utilization. All other questions are addressed by the authors.

We have changed the wording in the abstract (it was clear in the main text)
